# Rational design of a lipophilic β‐galactosidase‐responsive near‐infrared probe for in vivo imaging of cellular senescence

**DOI:** 10.1002/smo2.70091

**Published:** 2026-08-18

**Authors:** Jiani Huang, Feiyi Chu, Bin Feng, Lin Chen, Peili Cen, Jing Wang, Ying Qu, Lu Hong, Fei Wu, Huanfeng Tian, Yanpeng Fang, Yuanjie Chen, Mei Tian, Wenbin Zeng, Hong Zhang, Yan Zhong

**Affiliations:** ^1^ Department of Nuclear Medicine Zhejiang Cancer Hospital Hangzhou Institute of Medicine Chinese Academy of Sciences Hangzhou Zhejiang China; ^2^ Institute of Nuclear Medicine and Molecular Imaging of Zhejiang University Hangzhou Zhejiang China; ^3^ Key Laboratory of Medical Molecular Imaging of Zhejiang Province Hangzhou Zhejiang China; ^4^ Xiangya School of Pharmaceutical Sciences Central South University Changsha China; ^5^ Human Phenome Institute Fudan University Shanghai China

**Keywords:** β‐galactosidase, blood‐brain barrier, brain aging, cellular senescence, near‐infrared fluorescent probes, tumor senescence

## Abstract

Senescence‐associated β‐galactosidase (SA‐β‐gal) is a key biomarker of cellular senescence and has been demonstrated to be a major driver of various age‐related diseases and tumor resistance. However, noninvasive in vivo imaging of SA‐β‐gal remains challenging due to the poor membrane permeability of existing probes, their reliance on intratumoral injection, and limited blood‐brain barrier (BBB) permeability. This study reports a novel, rationally designed near‐infrared (NIR) fluorescent probe, **DCIP‐AcGal**, which involves a synergistic lipophilicity‐oriented design strategy by leveraging a BBB‐penetrating fluorophore with acetylated β‐galactose. Its SA‐β‐gal‐sensitive NIR emission property enables deep‐tissue imaging of senescent cells with low background and high signal‐to‐noise ratio. The probe's favorable lipophilicity facilitates passive diffusion through the cell membranes, ensuring efficient activation by SA‐β‐gal within the lysosomes. Its specificity for imaging senescent cells has been validated through GLB1 knockdown, and its fluorescence intensity and distribution correlate positively with p21 expression. Following intravenous injection, **DCIP‐AcGal** enables noninvasive imaging of palbociclib‐induced tumor senescence in living mice. Furthermore, due to its favorable lipophilicity, the probe crosses the BBB, allowing for the assessment of senescence accumulation in the brains of aged mice. These findings demonstrate that probe **DCIP‐AcGal** provides a valuable tool for investigating senescence biology and monitoring the efficacy of senotherapeutic interventions.

## Graphical abstract

We developed a lipophilic near‑infrared probe (**DCIP‑AcGal**) that is specifically activated by senescence‑associated β‑galactosidase. After intravenous injection, it lights up therapy‑induced tumor senescence in living mice. Its optimized lipophilicity also enables blood‑brain barrier crossing to detect natural brain senescence ex vivo. This probe offers a powerful tool for aging research and senotherapeutic monitoring.

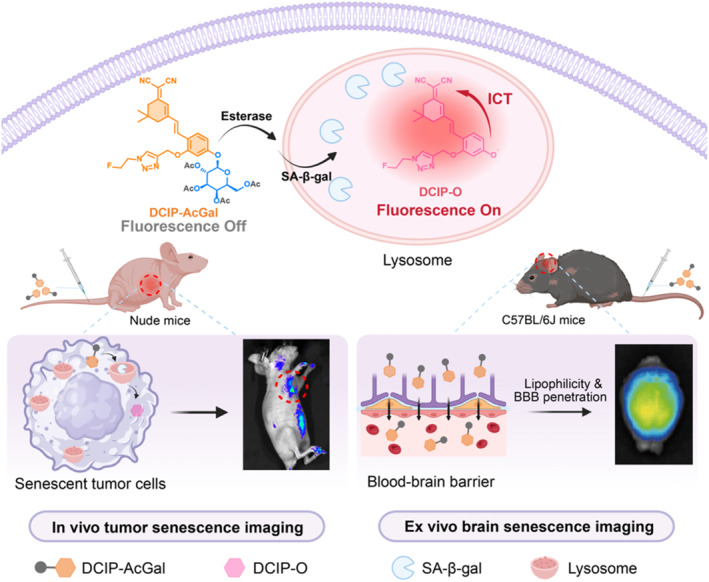

## 1 INTRODUCTION

Cellular senescence is a complex biological process characterized by a stable and irreversible arrest of cell proliferation, which can be triggered by various stresses including telomere dysfunction, DNA damage, oncogene activation, and oxidative stress.^[1]^ In addition to cell cycle arrest, cellular senescence is defined by several hallmark features, including activation of the DNA damage response, enhanced anti‐apoptotic signaling, induction of the senescence‐associated secretory phenotype, and a pronounced increase in lysosomal content.[^2^, ^3^] Among these features, senescence‐associated β‐galactosidase (SA‐β‐gal), a GLB1‐encoded lysosomal hydrolase,^[4]^ is highly enriched in the lysosomal compartment of senescent cells. As such, it has become the most widely accepted and thoroughly validated biomarker for senescence detection in cell culture and tissue samples.[^5^, ^6^] Therefore, sensitive and specific detection of SA‐β‐gal is critical not only for elucidating the biological mechanisms of aging but also for guiding therapeutic strategies against senescence‐associated diseases.^[7]^

Conventional SA‐β‐gal detection methods, such as X‐gal staining, require cell fixation, limiting their utility in longitudinal studies and real‐time monitoring of senescence dynamics in living organisms.^[8]^ In recent years, a large number of β‐gal‐activated fluorescent probes have been developed for the real‐time imaging and detection of cellular senescence.[^9^, ^10^, ^11^, ^12^, ^13^, ^14^] The core design principle involves using β‐galactose residues, substrates of SA‐β‐gal, to cage electron‐donating groups on the fluorophore.[^15^, ^16^] The resulting probes can regulate intramolecular charge transfer (ICT) through enzymatic hydrolysis, thereby achieving specific fluorescent activation in senescent cells.[^17^, ^18^, ^19^] Recent advances in probe engineering have further optimized probe performance, such as target affinity and signal retention, thereby advancing the development of molecular probes for detecting cellular senescence.[^20^, ^21^, ^22^]

Nevertheless, critical limitations still hinder the widespread clinical application of β‐gal‐activatable fluorescent probes for senescence imaging. From a molecular design perspective, a core flaw in existing probes is their insufficient affinity for phospholipid membrane: conventional probes directly conjugate the polyhydroxylated β‐galactose recognition moiety to the fluorophores.^[10]^ The strong hydrophilicity significantly reduces the overall lipophilicity of the probe, resulting in poor cell membrane permeability, which in turn severely compromises its in vivo distribution efficiency and imaging signal‐to‐noise ratio.[^23^, ^24^] Furthermore, most reported probes operate at short emission wavelengths, suffering from potential autofluorescence interference and limited tissue penetration depth, which compromise the sensitivity and accuracy of in vivo imaging in deep tissues.^[25]^ Most existing probes rely on localized administration routes, such as intratumoral injection for targeted imaging, which precludes the possibility of non‐invasive whole‐body detection of disseminated senescent lesions and significantly limits their clinical applicability in the assessment of systemic senescence burden.[^26^, ^27^, ^28^] More importantly, it's rare to consider the ability to cross the blood‐brain barrier (BBB) in the design of β‐gal‐activatable probes. Given that the abnormal accumulation of senescent cells in the central nervous system is a key pathological driver of age‐related neurodegenerative disorders such as Alzheimer's disease and Parkinson's disease,[^29^, ^30^] the development of senescence imaging probes with optimal BBB penetration has become an urgent unmet need in this field.[^31^, ^32^, ^33^]

**SCHEME 1:**
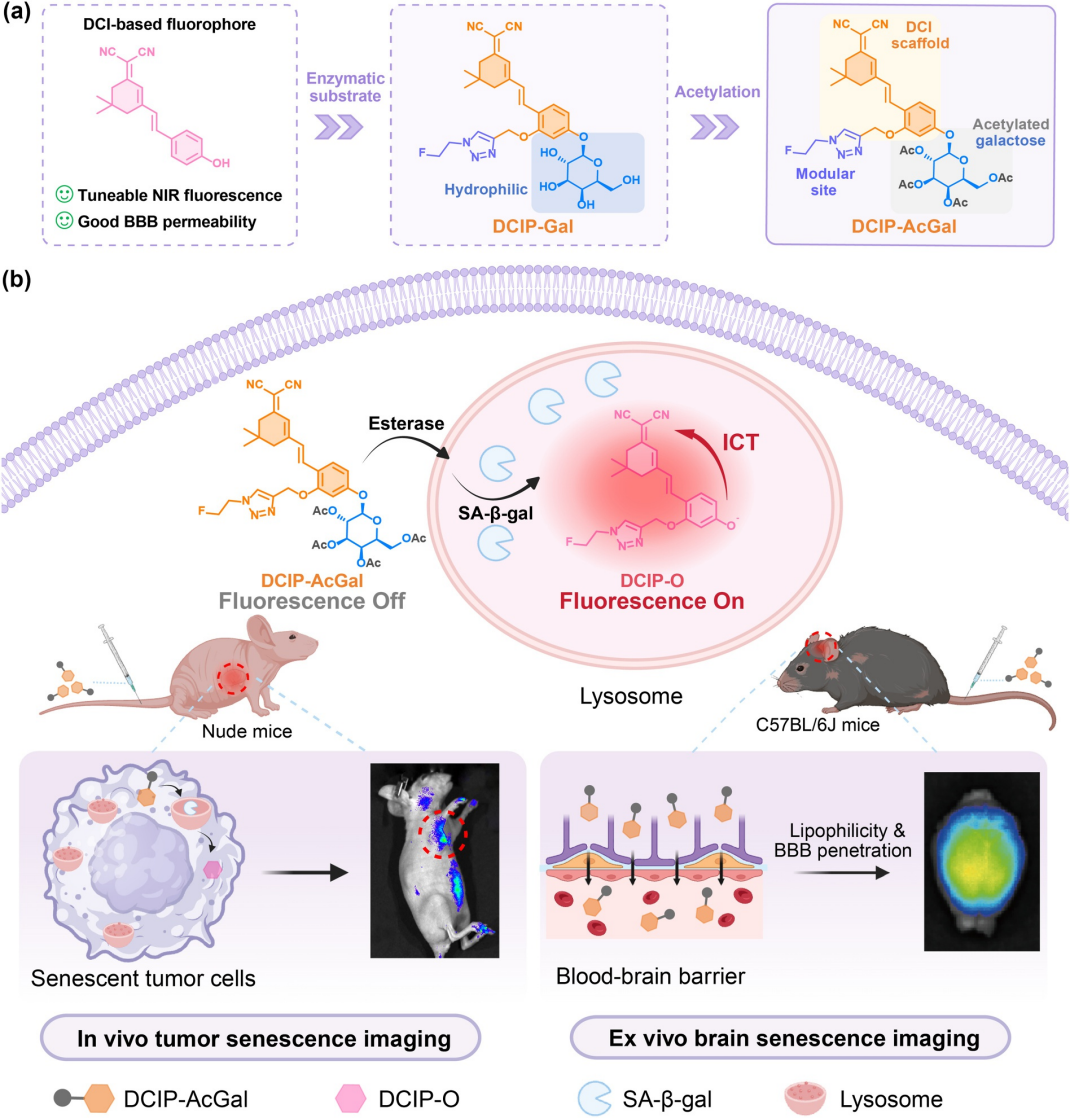
Schematic illustration of the NIR fluorescent probe **DCIP‐AcGal** for imaging cellular senescence. (a) Synergistic lipophilicity‐oriented design strategy for probe **DCIP‐AcGal** with SA‐β‐gal responsiveness. (b) Proposed sensing mechanism of **DCIP‐AcGal** for tumor senescence and natural brain senescence. NIR, near‐infrared; SA‐β‐gal, senescence‐associated β‐galactosidase.

To address these challenges, we herein report a novel, rationally designed near‐infrared (NIR) fluorescent probe for in vivo imaging of senescent cells (Scheme 1). A synergistic lipophilicity‐oriented design strategy was adopted for the first time to construct the lipophilic fluorescent probe **DCIP‐AcGal**, which utilizes acetylated β‐galactose as the recognition moiety for SA‐β‐gal and grafts it onto a fluorophore with BBB‐crossing capabilities. Its SA‐β‐gal‐sensitive NIR emission property enables deep‐tissue imaging of senescent cells with low background and high signal‐to‐noise ratio. Following intravenous administration, **DCIP‐AcGal** demonstrated excellent imaging performance in both chemotherapy‐induced tumor senescence models and the brain tissue of naturally aged mice, while exhibiting good biocompatibility. The novel NIR probe **DCIP‐AcGal** provides a robust tool for investigating the systemic senescence biology and for the early diagnosis of neurodegenerative diseases, while also opening new avenues for real‐time monitoring of the senotherapeutic efficacy in vivo.

## 2 RESULTS AND DISCUSSION

### 2.1 Rational design and synthesis of probe DCIP‐AcGal

The design of probe **DCIP‐AcGal** primarily involved a synergistic design strategy to optimize lipophilicity and BBB permeability. This probe employed a responsive NIR fluorescent reporter based on the dicyanoisophorone (**DCI**) scaffold as the signaling unit, which has been validated to possess good lipophilicity and potential BBB permeability.[^34^, ^35^, ^36^] On the other hand, an acetylated β‐galactose moiety was introduced as the SA‐β‐gal‐specific trigger. The peracetylation of all free hydroxyl groups of the β‐galactose unit significantly reduces the hydrophilicity of the sugar moiety, ensuring the overall lipophilicity of **DCIP‐AcGal** to facilitate passive diffusion across the cell membrane. Utilizing this lipophilicity‐oriented synergistic design, the probe could efficiently penetrate senescent cells and be specifically recognized and cleaved by SA‐β‐gal in lysosomes, releasing the fluorophore with restored NIR fluorescence enhancement. Detection based on the enzyme‐catalyzed fluorescent signal enables non‐invasive imaging and specific localization of senescent cells in various disease models, including the imaging of senescent cells in the brain. Notably, the incorporated fluoroethyl azide does not participate in the SA‐β‐gal‐specific recognition, instead it provides a convenient modular synthetic site for future fluorine‐18 radiolabeling to meet the application requirements of positron emission tomography (PET) imaging in multimodal senescence imaging.[^37^, ^38^]

The probe **DCIP‐AcGal** was synthesized via a streamlined, five‐step route (Supporting Information S1: Scheme S1). Initially, the **DCI** fluorophore was obtained via the Knoevenagel condensation of isophorone and malononitrile. Subsequently, a β‐galactopyranosidic bond was formed via a nucleophilic substitution reaction, and **DCI** was coupled to the intermediate via a Knoevenagel condensation to yield an acetylated intermediate. Then, a terminal alkyne handle was introduced by propargylation of the phenolic hydroxyl group with propargyl bromide. Finally, a copper(II)‐catalyzed azide‐alkyne cycloaddition reaction was carried out between the alkyne group and the in situ generated 1‐azido‐2‐fluoroethane, ultimately yielding probe **DCIP‐AcGal**. All synthesized compounds and intermediates were rigorously characterized using ^1^H nuclear magnetic resonance (NMR), ^13^C NMR, and high‐resolution mass spectrometry (HRMS) (Supporting Information S1: Figures S1–S7).

### 2.2 Spectroscopic response to β‐gal activity

**FIGURE 1:**
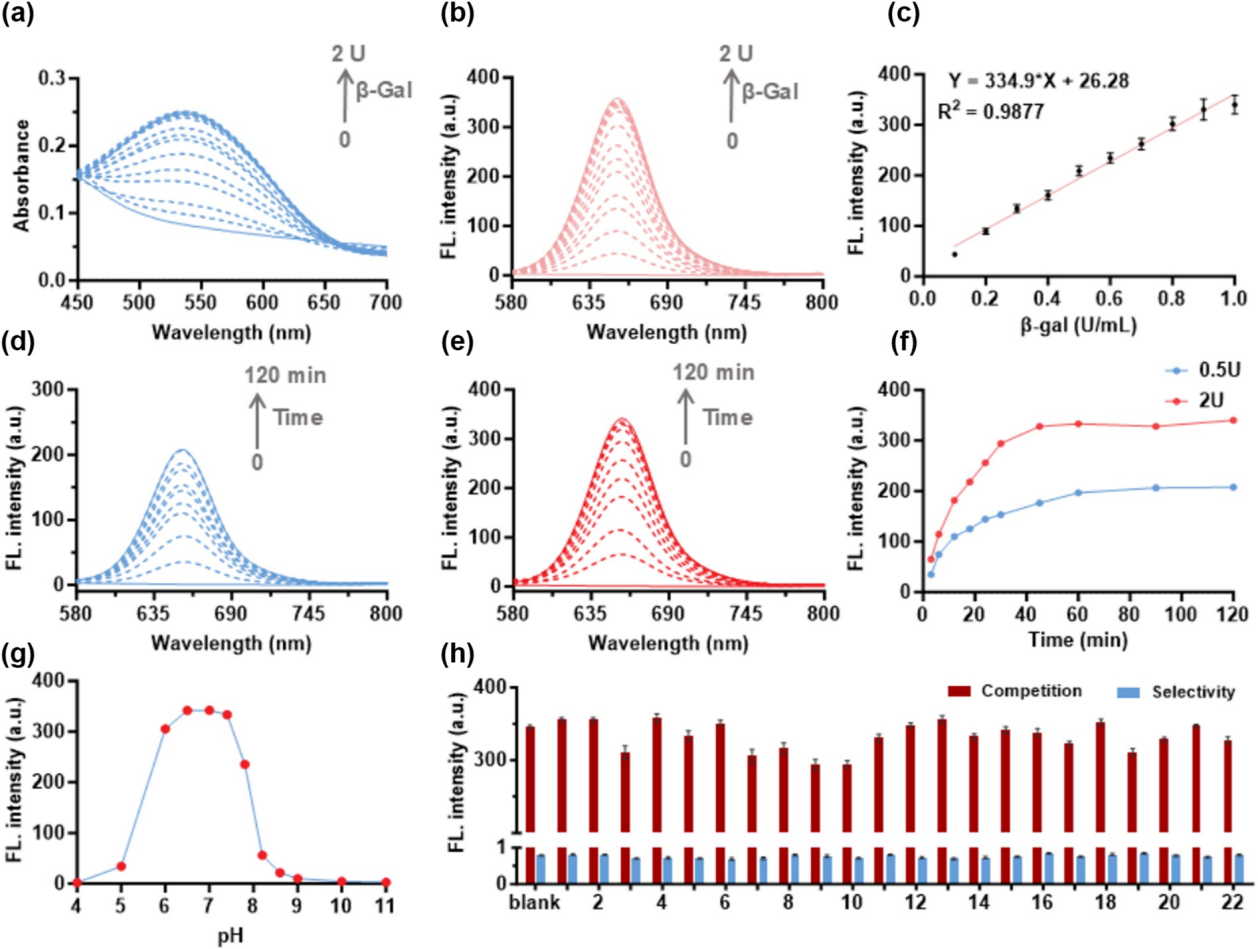
Evaluations of the response of **DCIP‐AcGal** to β‐gal. (a, b) Absorption spectra (a) and fluorescence emission spectra (b) of **DCIP‐AcGal** (10 μM) upon incubation with β‐gal (0–2 U/mL) for 60 min, respectively. (c) Linear relationship between fluorescence intensity of **DCIP‐AcGal** (10 μM) at 657 nm and β‐gal concentration. (d, e) Time‐dependent fluorescence emission spectra of **DCIP‐AcGal** (10 μM) upon incubation with β‐gal (0.5 U/mL, d) and β‐gal (2 U/mL, e), respectively. (f) Kinetic time curve of **DCIP‐AcGal** (10 μM) incubated with β‐gal (0.5 U/mL and 2 U/mL). (g) Fluorescent intensity of **DCIP‐AcGal** (10 μM) after incubation with β‐gal (2 U/mL) at different pHs (4.0–11.0). (h) Fluorescent intensity of **DCIP‐AcGal** (10 μM) upon incubation with various interferents in the presence/absence of β‐gal (2 U/mL) for 45 min. Interferents: 1–6 (100 μm Na_2_CO_3_, FeSO_4_, FeCl_3_, KH_2_PO_4_, MgCl_2_, CaCl_2_), 7–9 (100 μm Cys, Hcy, GSH), 10–16 (1 U/mL ALP, GGT, CE, NTR, APN, Try, Chymotrypsin), 17–22 (100 μm H_2_O_2_, HClO, BSA, heparin, glucose, ribose). *λ*_ex_ = 540 nm.

Having obtained the probe, a titration assay was first conducted to evaluate the optical response of **DCIP‐AcGal** to β‐gal in phosphate buffer saline buffer (30% dimethyl sulfoxide, pH 7.4). As illustrated in Figure 1a,b, the probe exhibited negligible absorption at 540 nm and weak fluorescence emission at 657 nm, indicative of its low background signal. Upon incubation with β‐gal, both the absorbance and the fluorescence intensity exhibited a dose‐dependent, progressive enhancement. Notably, a good linear relationship was found between the fluorescence intensity at 657 nm and the concentration of β‐gal within the range of 0–2 U/mL (*y* = 334.90*x* + 26.28, *R* ^2^ = 0.9877, Figure 1c). The limit of detection was determined to be 0.059 U/L (3*σ*/*k*), demonstrating the feasibility of **DCIP‐AcGal** for the sensitive and quantitative detection of β‐gal activity. The kinetic profile of **DCIP‐AcGal**'s response toward β‐gal was further investigated. As shown in Figure 1f, the fluorescence intensity increased rapidly upon exposure to β‐gal and reached a plateau within 45 min (0.5 U/mL, Figure 1d) or 60 min (2 U/mL, Figure 1e). Photostability assays showed that, after 1 h of continuous irradiation, the activated fluorescence signal exhibited good stability (attenuation rate <8%) (Supporting Information S1: Figure S8), while the unactivated probe maintained a consistently low background, indicating its suitability for long‐term monitoring applications. Moreover, the fluorescence intensity of the β‐gal‐triggered probe remained stable over a broad pH range of 6.0–8.0 (Figure 1g). Of note, the lysosomal compartments in senescent cells have a characteristic pH of 6, within which **DCIP‐AcGal** still maintained a strong fluorescent response.[^39^, ^40^] Given the complexity of biological matrices, the specificity of **DCIP‐AcGal** for β‐gal was further assessed in the presence of various potential interferents, including ions (Na_2_CO_3_, FeSO_4_, FeCl_3_, KH_2_PO_4_, MgCl_2_, CaCl_2_), amino acids (Cys, Hcy, GSH), enzymes (ALP, GGT, CE, NTR, APN, Try, Chymotrypsin), and bioactive molecules (H_2_O_2_, HClO, BSA, heparin, glucose, ribose). As shown in Figure 1h, negligible fluorescence was triggered upon exposure to these interferents, which also had no significant effect on the fluorescence enhancement caused by β‐gal hydrolyzing **DCIP‐AcGal**. Although the esterase present could hydrolyze the acetyl groups in **DCIP‐AcGal**, it could not affect the galactosidic bond and was therefore unable to influence the corresponding changes in fluorescence. These results demonstrated the applicability of **DCIP‐AcGal** for β‐gal detection in complex biological samples.

### 2.3 Sensing mechanism of probe DCIP‐AcGal

**FIGURE 2:**
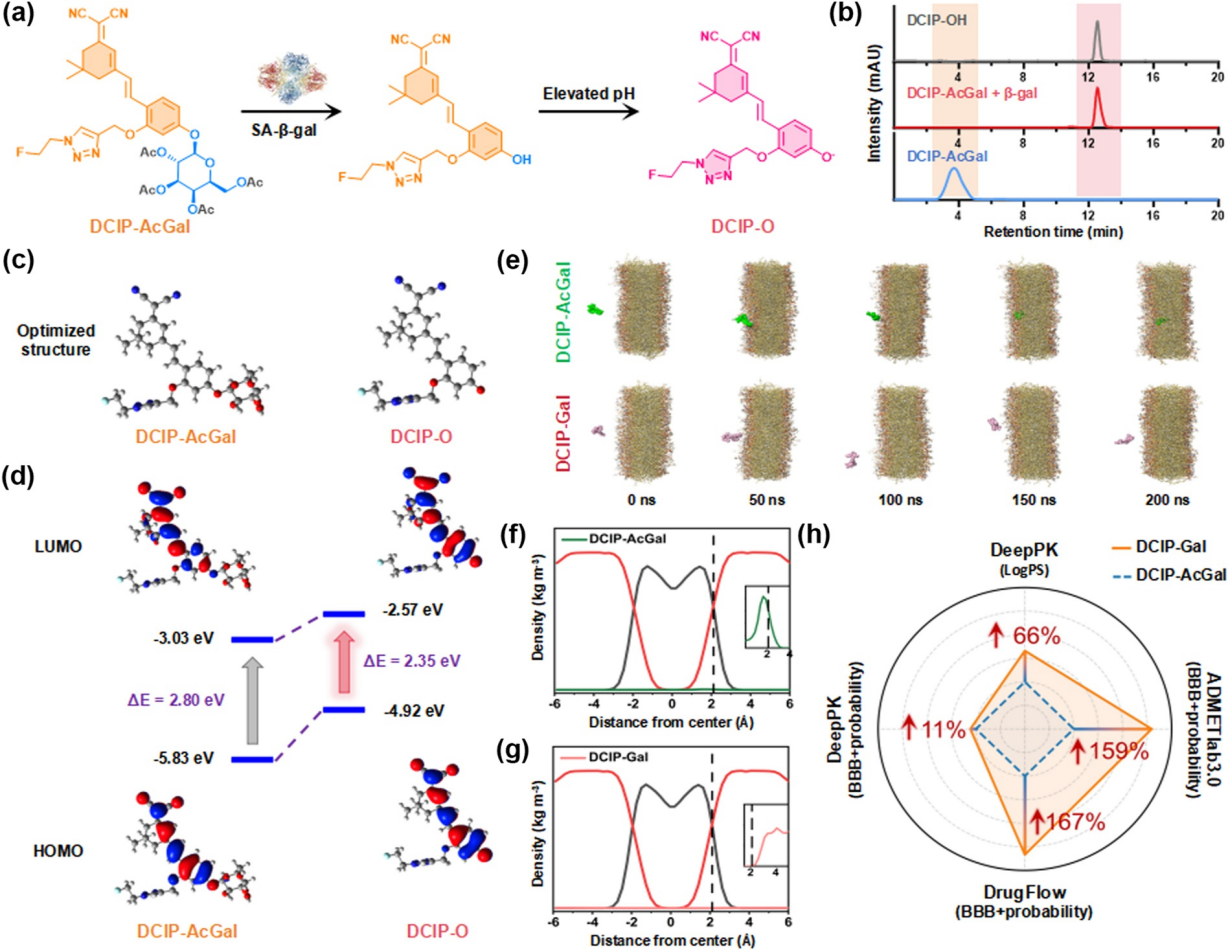
Sensing mechanism of probe **DCIP‐AcGal** for SA‐β‐gal. (a) Molecular mechanism of β‐gal‐triggered **DCIP‐AcGal**. (b) HPLC analysis of fluorophore **DCIP‐OH**, probe **DCIP‐AcGal**, and **DCIP‐AcGal** incubated with β‐gal. (c, d) Optimized molecular structure (c) and frontier molecular orbital (d) of **DCIP‐AcGal** and **DCIP‐OH**. (e) Snapshots from MD simulations of **DCIP‐AcGal** (green) and **DCIP‐Gal** (pink) within the phospholipid bilayer system. (f, g) Mass density profile of **DCIP‐AcGal** (f) and **DCIP‐Gal** (g) during simulations. Red and black curves represent water and phospholipid bilayer mass density profiles, respectively. Insert: the main distribution of the corresponding probe at the water‐phospholipid bilayer interface (2 nm). (h) Evaluation of the BBB permeability of **DCIP‐AcGal** and **DCIP‐Gal** using three online computational platforms (ADMETlab3.0, DeepPK, and DrugFlow). BBB, blood‐brain barrier; HPLC, high‐performance liquid chromatography; MD, molecular dynamics; SA‐β‐gal, senescence‐associated β‐galactosidase.

To validate the enzymatic role in the fluorescence activation, competitive inhibition assays were first conducted. As shown in Supporting Information S1: Figure S9, the presence of the inhibitor D‐galactose inhibited enzyme‐mediated fluorescence enhancement, and the signal intensity exhibited a dose‐dependent decrease with increasing inhibitor concentration, indicating that β‐gal activity is critical to the probe's optical response. Notably, 1 mM D‐galactose caused an inhibition of up to 79%. This high inhibition efficiency was partly attributed to the fact that D‐galactose, with its exposed hydroxyl group, has a higher affinity for the enzymatic pocket compared to the acetylated substrate on **DCIP‐AcGal**. Next, the molecular mechanism underlying the enzymatic activation process was monitored through high‐performance liquid chromatography. The retention times of **DCIP‐AcGal** and **DCIP‐OH** were 3.70 and 12.54 min, respectively. Upon incubation with β‐gal, the peak of **DCIP‐AcGal** at 3.70 min was markedly reduced, while another peak emerged at 12.54 min, consistent with the retention time of **DCIP‐OH** (Figure 2b). Further analysis of HRMS results showed that upon activation by β‐gal, the mass peak of **DCIP‐AcGal** shifted from m/z 726.2947 to m/z 432.1848 (Supporting Information S1: Figure S10), which matched well with the theoretical mass of the fluorophore **DCIP‐OH** (calcd. m/z 432.1836). To rationalize the fluorescence turn‐on response, frontier molecular orbital analysis was performed. As shown in Figure 2c,d, the highest occupied molecular orbital‐lowest unoccupied molecular orbital energy gap of the released fluorophore **DCIP‐OH** (2.35 eV) was smaller than that of **DCIP‐AcGal** (2.80 eV). This reduction in energy gap accounted for the observed bathochromic shift in absorption and emission spectra, which could be attributed to the restoration of the ICT process upon enzymatic cleavage. These results collectively demonstrated that β‐gal specifically hydrolyzed the glycosidic bond in **DCIP‐AcGal**, leading to the release of the fluorophore and subsequent deprotonation of the hydroxyl group under physiological conditions; ultimately, this resulted in enhanced ICT strength, manifested as a “Turn‐On” NIR fluorescence response (Figure 2a). It was noteworthy that the slow activation of **DCIP‐AcGal** observed could be attributed to the steric hindrance imposed by the peracetylation of galactose moiety.[^41^, ^42^] This structural modification partially compromises the binding affinity of **DCIP‐AcGal** to β‐gal, thereby retarding the rate of enzymatic cleavage and the subsequent release of the fluorophore. Nevertheless, within senescent cells, an alternative and highly efficient activation mechanism coexists alongside direct β‐gal hydrolysis (Supporting Information S1: Scheme S2). Specifically, **DCIP‐AcGal** can undergo deacetylation mediated by endogenous esterases, which are abundant inside cells, generating the glycosylated intermediates. These unmasked intermediates are subsequently recognized and rapidly cleaved by β‐gal, resulting in the activation of the NIR fluorescence signal.

To further investigate the phospholipid membrane penetration properties of **DCIP‐AcGal**, molecular dynamics simulations were performed to analyze the membrane affinity. As shown in Figure 2e, **DCIP‐AcGal** rapidly anchored to the membrane surface and progressively embedded in the phospholipid bilayer during the 200 ns simulation. In contrast, **DCIP‐Gal** failed to form stable binding with the bilayer. Quantitative analysis of mass density profiles (Figure 2f,g) revealed that **DCIP‐AcGal** was primarily distributed left of the water‐POPC interface (2 nm), indicating tight association with the phospholipid membrane. Conversely, **DCIP‐Gal** was distributed to the right of the interface, demonstrating its solvation in the aqueous phase. This was further supported by the centroid distance analysis (Supporting Information S1: Figures S11 and S12). Additionally, root‐mean‐square deviation (RMSD) trajectories showed that, unlike the persistent fluctuations in **DCIP‐Gal**, **DCIP‐AcGal** exhibited reduced conformational fluctuations following membrane stabilization (Supporting Information S1: Figures S13 and S14). These results demonstrated that acetylated **DCIP‐AcGal** exhibited superior phospholipid membrane affinity than **DCIP‐Gal**. Given the increased lipophilicity, we expected that the acetylated **DCIP‐AcGal** would also exhibit improved BBB permeability. Three online computational platforms (ADMETlab 3.0, DeepPK, and DrugFlow) were utilized to predict the BBB permeability of probes **DCIP‐Gal** and **DCIP‐AcGal**, respectively.[^43^, ^44^, ^45^] These platforms provided complementary in silico assessments of BBB transport by reporting both probability‐based classification outputs and a permeability‐related continuous descriptor. Due to the hydrophilicity of β‐galactose, the prediction results for **DCIP‐Gal** were unsatisfactory across all platforms. In contrast, **DCIP‐AcGal**, which contains an acetylated substrate, consistently showed increased predicted BBB permeability across all readouts due to its enhanced lipophilicity (Figure 2h). Although the absolute values from these models were not directly comparable because they represented different categories of predictive outputs, the concordant direction of change across the three platforms supported the conclusion that acetylation improved the probe's BBB permeability.

### 2.4 Specific activation and subcellular localization of DCIP‐AcGal as a senescence probe

**FIGURE 3:**
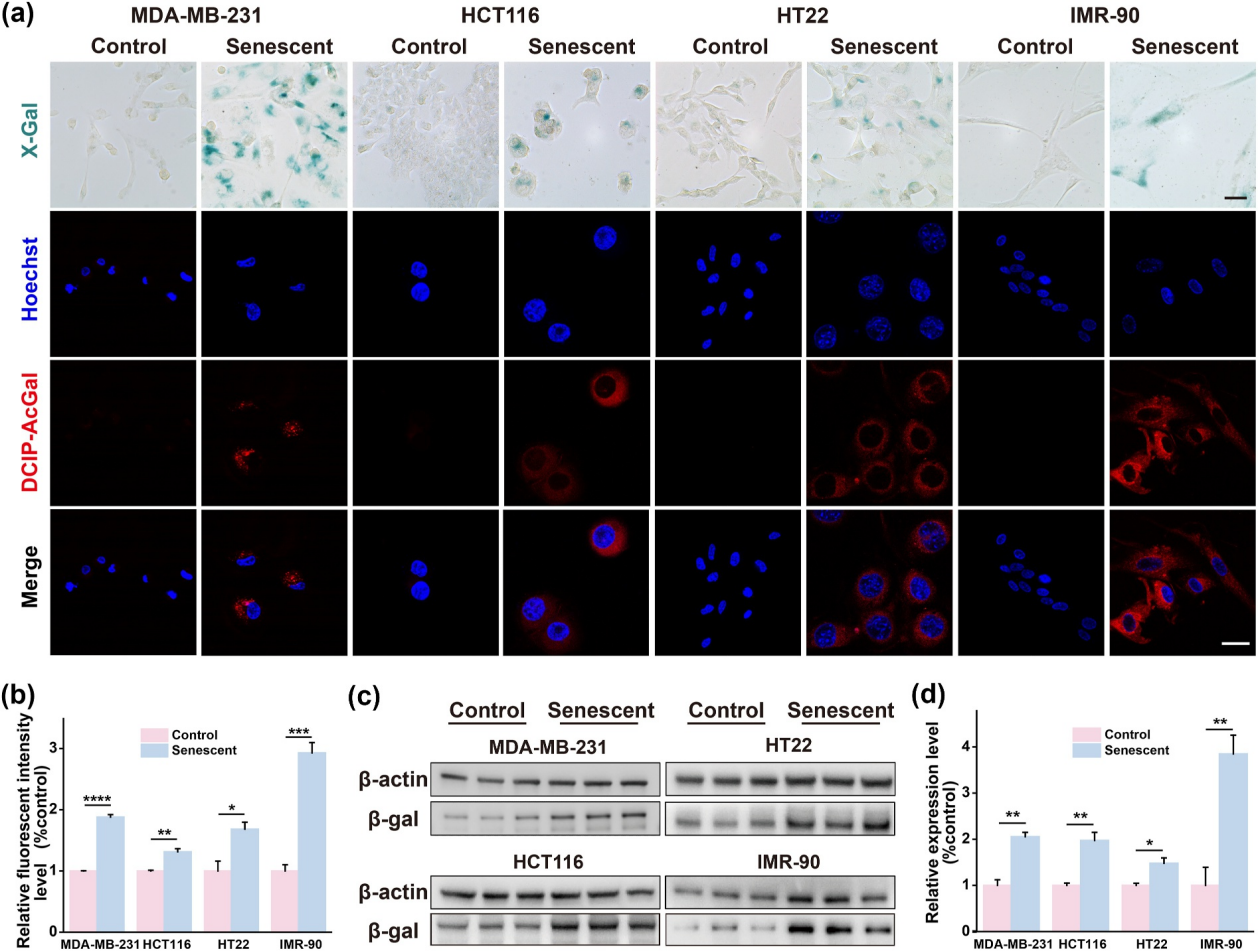
Validation of cellular senescence models and the application of **DCIP‐AcGal** as a SA‐β‐gal probe. (a) X‐gal staining of control and senescent MDA‐MB‐231, HCT116, HT22 and IMR‐90 cells (scale bar, 50 μm) and fluorescence imaging stained with **DCIP‐AcGal** (scale bar, 30 μm). Nuclei were counterstained with Hoechst 33342 (blue). (b) Semiquantitative analysis of mean **DCIP‐AcGal** fluorescent intensity in (a) (*n* = 3). (c) Western blotting analysis of β‐gal in MDA‐MB‐231, HCT116, HT22 and IMR‐90 cells. (d) Semiquantitative analysis of β‐gal protein expression in (c) (*n* = 3). (Statistical significance was determined using a two‐tailed unpaired Student's *t*‐test. *p < 0.05; **p < 0.01; ***p < 0.001; ****p < 0.0001). SA‐β‐gal, senescence‐associated β‐galactosidase.

Building on its favorable in vitro properties, we next evaluated the applicability of **DCIP‐AcGal** for detecting β‐gal activity in different types of senescent cells. Tumor cell senescence models were established in MDA‐MB‐231 and HCT116 cells using doxorubicin induction,^[46]^ while normal cell senescence models were generated in HT22 cells via bleomycin treatment and in IMR‐90 cells through replicative senescence.[^47^, ^48^] As shown in Figure 3a, X‐gal staining revealed intense blue‐green coloration in senescent cells across all four cell lines, whereas control cells exhibited minimal signal, confirming successful senescence induction. Consistent with these observations, Western blotting analysis demonstrated an approximately two‐fold increase in β‐gal protein expression in senescent cells compared with their respective controls (Figure 3c,d), further validating the establishment of senescence models.

Prior to applying **DCIP‐AcGal** to cells, its potential cytotoxicity was assessed using the CCK‐8 assay. As illustrated in Supporting Information S1: Figure S15A–D, after 24 h of **DCIP‐AcGal** treatment, all four types of control and senescent cells maintained over 90% cell viability, demonstrating the excellent biocompatibility of the probe. Following addition of **DCIP‐AcGal** to the culture medium, fluorescence imaging revealed robust red fluorescence signals in senescent MDA‐MB‐231 and HCT116 cells (Figure 3a,b), while control cells showed negligible fluorescence. Flow cytometric analysis further corroborated these findings, with senescent cells exhibiting markedly increased fluorescence intensity, approximately 2‐fold in MDA‐MB‐231 cells and 4‐fold in HCT116 cells compared to the control (Supporting Information S1: Figure S16A,B), indicating enhanced probe retention and β‐gal specific activation. Consistent results were obtained in normal cell senescence models, with HT22 and IMR‐90 cells displaying significantly enhanced fluorescence upon **DCIP‐AcGal** incubation (Figure 3a,b).

**FIGURE 4:**
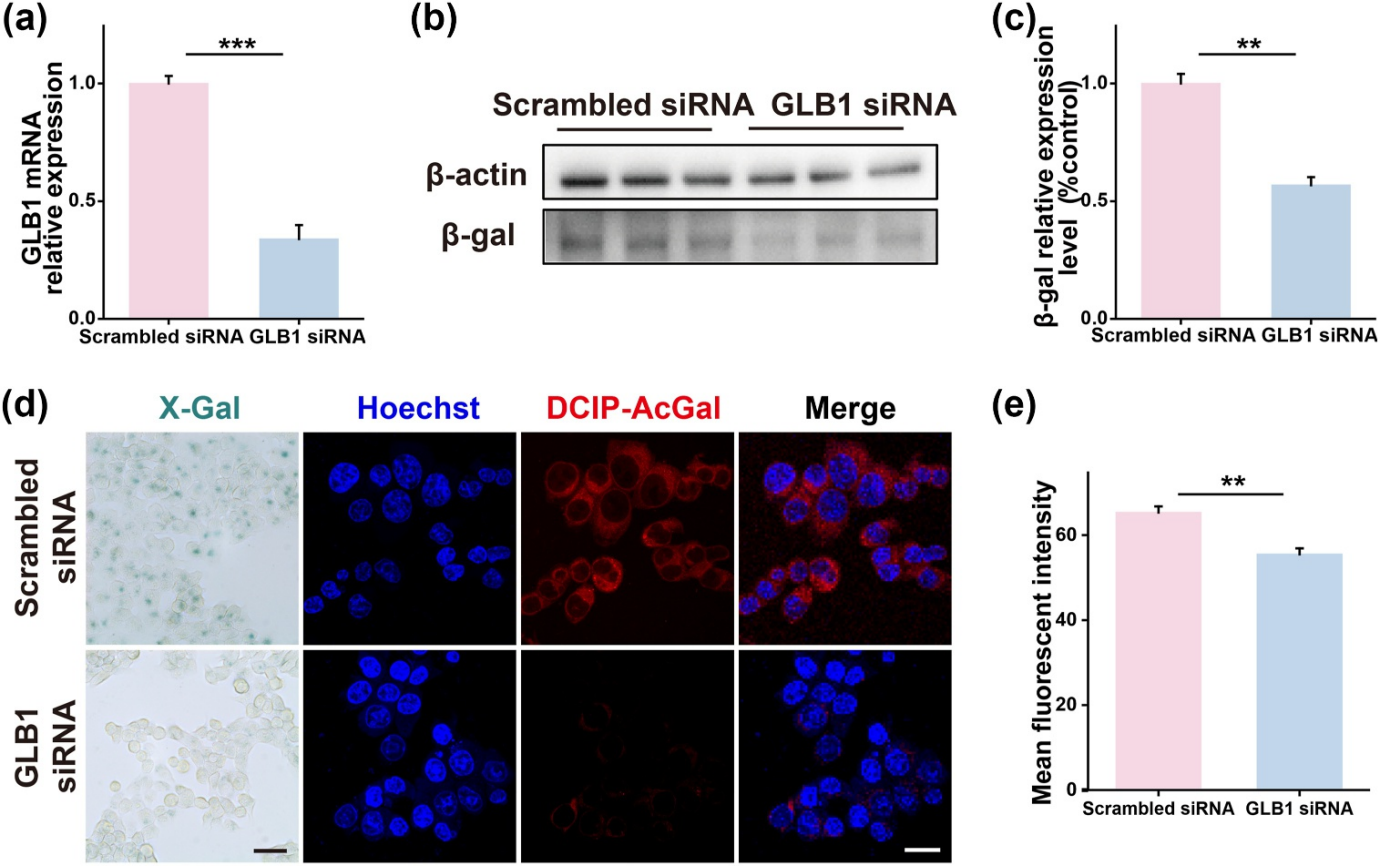
Specific activation of **DCIP‐AcGal** for β‐gal in senescent cells. (a) mRNA expression level of GLB1 in senescent HCT116 cells after GLB1 knockdown. (b) Western blotting analysis and (c) semiquantitative analysis of β‐gal protein expression in senescent HCT116 cells after GLB1 knockdown. (d) X‐gal staining (scale bar, 50 μm) and **DCIP‐AcGal** fluorescence imaging of senescent HCT116 cells after GLB1 knockdown (scale bar, 30 μm). Nuclei were counterstained with Hoechst 33342 (blue). (e) Semiquantitative analysis of mean **DCIP‐AcGal** fluorescent intensity in (d) (*n* = 3). (Statistical significance was determined using a two‐tailed unpaired Student's *t*‐test. **p < 0.01; ***p < 0.001).

To validate the specificity of **DCIP‐AcGal** for β‐gal in senescent cells, we performed GLB1 knockdown experiments in senescent HCT116 cells using targeted siRNA against *GLB1*, the gene encoding β‐gal. As demonstrated in Figure 4a–d, transfection with *GLB1* siRNA effectively reduced both *GLB1* mRNA and protein expression compared to the scrambled siRNA control, which correlated with diminished SA‐β‐gal activity as evidenced by fainter X‐gal staining. Crucially, when **DCIP‐AcGal** was applied to *GLB1*‐silenced senescent HCT116 cells, the probe signal was drastically attenuated (Figure 4d,e), confirming that **DCIP‐AcGal** activation was specifically dependent on β‐gal activity. Collectively, these results established **DCIP‐AcGal** as a reliable fluorescent probe for detecting senescent cells via SA‐β‐gal, demonstrating its applicability across multiple senescence models and its specificity for β‐gal‐mediated activation.

**FIGURE 5:**
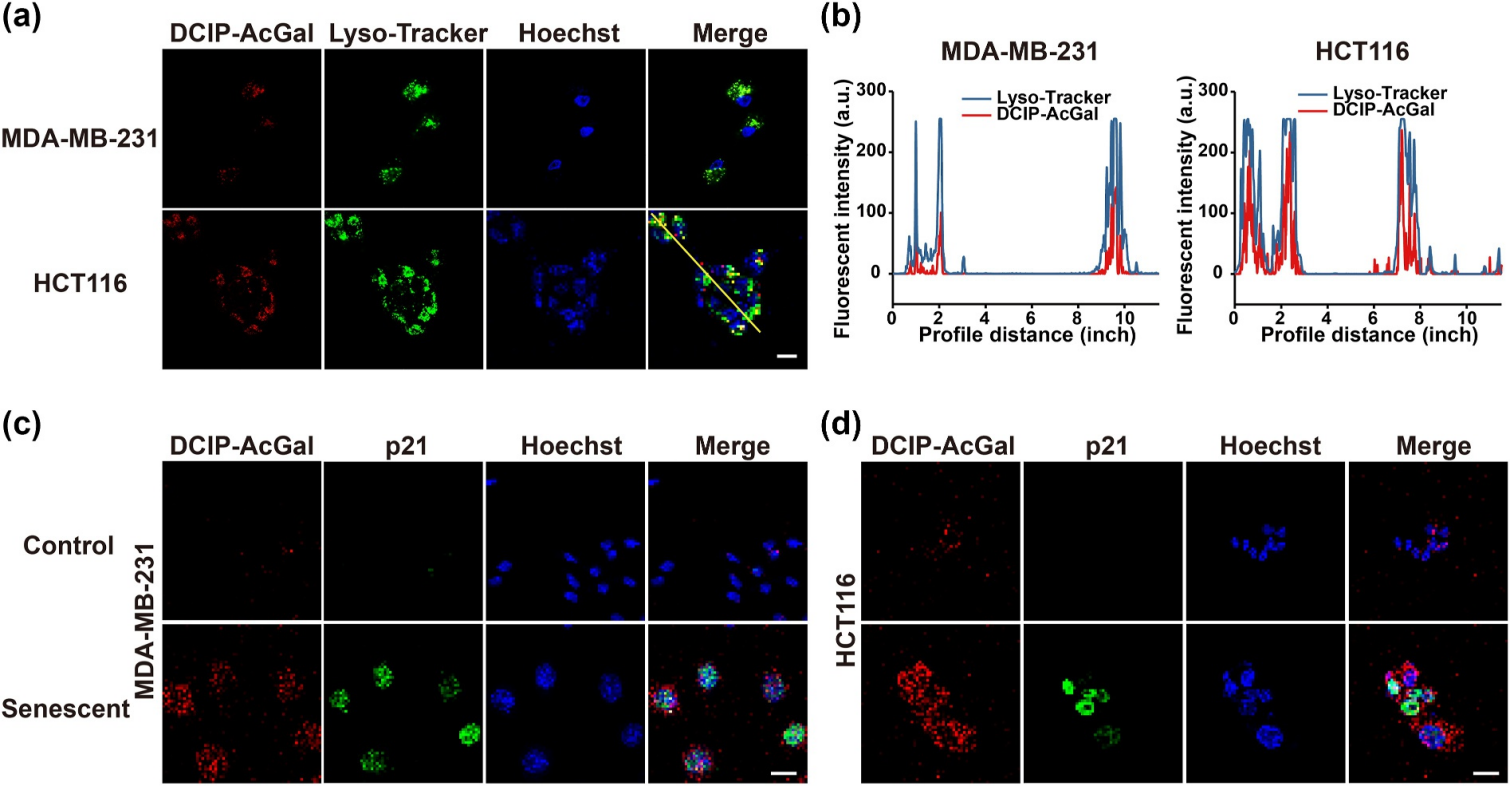
Lysosomal localization of **DCIP‐AcGal** and its correlation with p21 expression in senescent cells. (a) Fluorescence imaging of senescent MDA‐MB‐231 and HCT116 cells co‐stained with **DCIP‐AcGal** (red) and LysoTracker (green). Nuclei were counterstained with Hoechst 33342 (blue). Scale bar, 20 μm. (b) Fluorescence intensity profiles of **DCIP‐AcGal** and Lyso Tracker along the yellow lines depicted in (a). Immunofluorescence images of senescent (c) MDA‐MB‐231 and (d) HCT116 cells stained with **DCIP‐AcGal** (red) and p21 (green). Nuclei were counterstained with DAPI (blue). Scale bar, 20 μm. DAPI, 4',6‐diamidino‐2‐phenylindole.

To gain deeper insight into the subcellular distribution of **DCIP‐AcGal** and its relationship with β‐gal activity, we conducted colocalization studies using the lysosome‐specific tracker Lyso‐Tracker in senescent cells. As shown in Figure 5a, fluorescence imaging of senescent MDA‐MB‐231 and HCT116 cells co‐stained with **DCIP‐AcGal** (red) and Lyso‐Tracker (green) revealed excellent colocalization. The **DCIP‐AcGal** fluorescence exhibited a punctate pattern resembling the typical morphology of lysosomes, with substantial yellow signals observed in the merged images, indicating a significant overlay between the two channels. To quantitatively assess this colocalization, fluorescence intensity profiles were generated along the regions indicated by the yellow lines in Figure 5a. As presented in Figure 5b, the distribution curves of **DCIP‐AcGal** and Lyso‐Tracker were closely aligned, with Pearson's correlation coefficients of 0.85 and 0.65 for MDA‐MB‐231 and HCT116 senescent cells, respectively. These values confirmed that the majority of **DCIP‐AcGal** signals originated from lysosomal compartments, demonstrating that its response to β‐gal occurred specifically within lysosomes. This observation was consistent with the established biology of SA‐β‐gal, which accumulates in lysosomes during cellular senescence.

Having established the lysosomal localization of **DCIP‐AcGal**, we next sought to correlate its activation with canonical senescence markers at the single‐cell level. Cells were incubated with **DCIP‐AcGal** followed by immunostaining for p21, a well‐characterized cell cycle inhibitor and senescence regulator that accumulated in the nucleus of senescent cells.^[49]^ As illustrated in Figure 5c,d, senescent MDA‐MB‐231 and HCT116 cells displayed intense red fluorescence after **DCIP‐AcGal** incubation, which coincided with markedly enhanced nuclear p21 signals. The concomitant upregulation of both markers in individual senescent cells demonstrated that **DCIP‐AcGal** activation corresponded with the expression of established senescence regulators, validating its reliability as a senescence‐associated probe.

### 2.5 In vivo imaging of tumor senescence using DCIP‐AcGal

**FIGURE 6:**
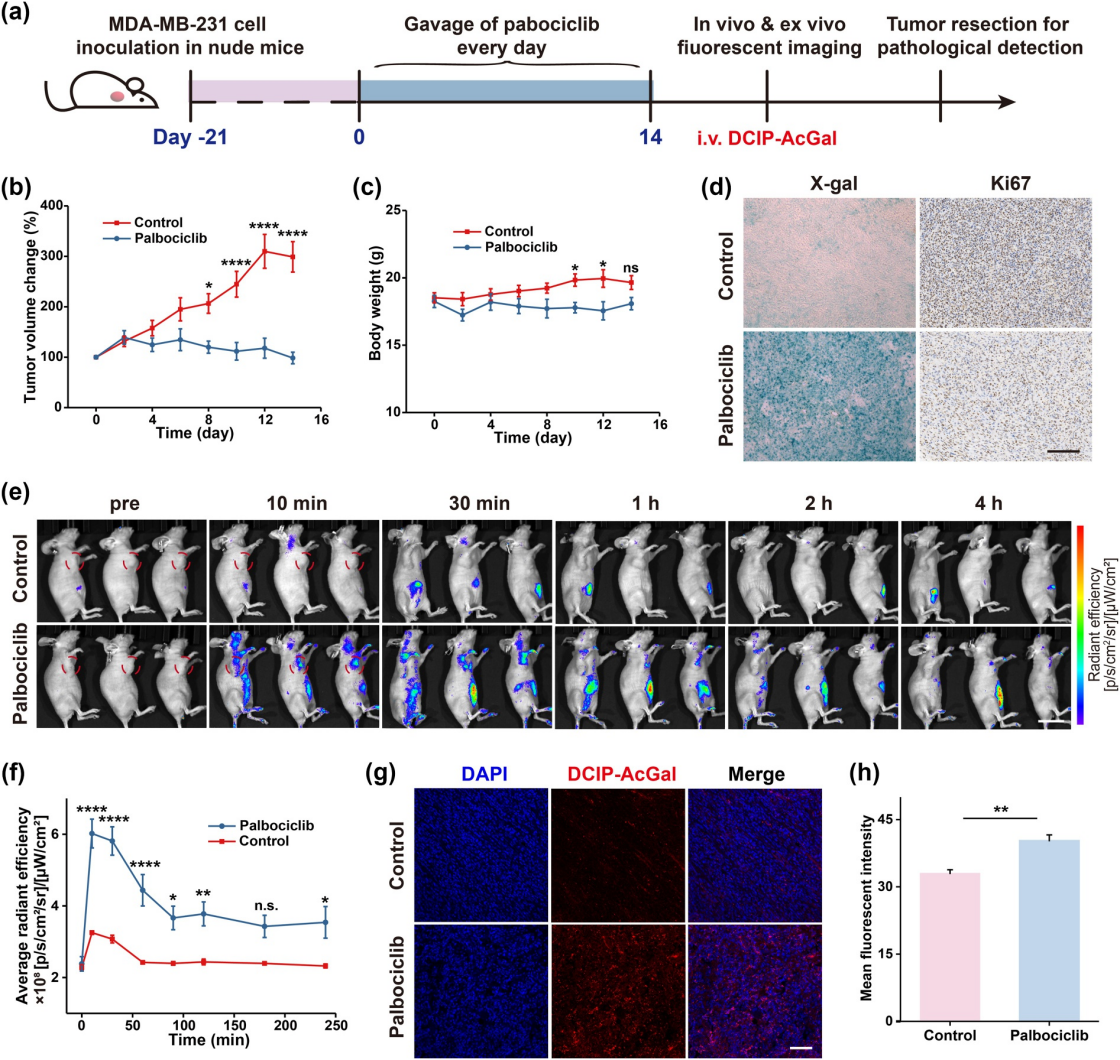
In vivo imaging of palbociclib‐induced tumor senescence using **DCIP‐AcGal**. (a) Schematic illustration of the tumor senescence modeling procedure and experimental timeline. (b) Tumor volume and (c) body weight changes in control and palbociclib‐treated mice during the treatment period (*n* = 6). (d) X‐gal and Ki67 staining of excised tumor sections of control and palbociclib‐treated tumors. Scale bar, 200 μm. (e) In vivo fluorescence imaging of control and palbociclib‐treated tumor‐bearing mice after intravenous injection of **DCIP‐AcGal** at pre‐injection, 10 min, 30 min, 1 h, 2 h, and 4 h post‐injection. The red circled region indicates tumor tissues. Scale bar, 2 cm. (f) Quantification of tumor fluorescence intensities from in vivo imaging at indicated time points (*n* = 3). (g) Representative fluorescence imaging of tumor cryosections from control and palbociclib‐treated mice after intravenous injection with **DCIP‐AcGal** (red), and counterstained with DAPI (blue). Scale bar, 100 μm. (h) Semiquantitative analysis of mean **DCIP‐AcGal** fluorescent intensity in (g) (*n* = 3). (Statistical significance was determined using a two‐tailed unpaired Student's *t*‐test. ns: not significant; *p < 0.05; **p < 0.01; ****p < 0.0001). DAPI, 4',6‐diamidino‐2‐phenylindole.

Given the promising performance of **DCIP‐AcGal** in detecting senescent cells in vitro, we next investigated its applicability for noninvasive imaging of therapy‐induced tumor senescence in living mice. A xenograft tumor model was established by inoculating MDA‐MB‐231 cells into nude mice, and tumor senescence was induced by daily oral gavage of palbociclib, a selective CDK4/6 inhibitor known to induce cellular senescence.[^50^, ^51^] The experimental timeline and procedure are illustrated in Figure 6a. During the treatment period, we monitored tumor volume and body weight changes in both control and palbociclib‐treated groups. As shown in Figure 4b, palbociclib treatment significantly suppressed tumor growth compared to the control group, consistent with its cytostatic effect. A slight decrease in body weight was observed in palbociclib‐treated mice (Figure 6c), likely attributable to the treatment regimen. To confirm the successful establishment of tumor senescence, excised tumors were subjected to X‐gal and Ki67 staining (Figure 6d). Palbociclib‐treated tumors exhibited intense SA‐β‐gal staining, indicating elevated β‐gal activity, while Ki67‐positive proliferative cells were markedly reduced compared with control tumors. These results confirm that palbociclib treatment effectively induced senescence in MDA‐MB‐231 xenografts.

We next evaluated the ability of **DCIP‐AcGal** to track tumor senescence burden in vivo. Following intravenous injection of the probe, fluorescence imaging was performed at various time points. As shown in Figure 6e, palbociclib‐treated mice displayed progressively increasing fluorescence signals in the tumor region, reaching peak intensity at approximately 30 min post‐injection and maintaining substantial target retention at 1 h. In contrast, control mice showed minimal tumor‐associated fluorescence throughout the imaging period. Quantitative analysis of tumor fluorescence intensity (Figure 6f) confirmed significantly higher signals in the palbociclib‐induced senescence group compared to controls, demonstrating that **DCIP‐AcGal** can specifically visualize tumor senescence with high β‐gal activity in living animals. To further validate the tumor‐specific accumulation of **DCIP‐AcGal**, ex vivo fluorescence imaging of major organs and tumors was performed 30 min post‐injection (Supporting Information S1: Figure S17A). Consistent with the in vivo biodistribution results, excised tumors from palbociclib‐treated mice exhibited markedly stronger fluorescence than those from control mice. Quantification of ex vivo fluorescence signals (Supporting Information S1: Figure S17B) revealed a more than 2‐fold increase in the senescence group compared to controls, while no substantial differences were observed in other organs between the two groups, indicating the tumor‐specific detection of **DCIP‐AcGal**. To corroborate these macroscopic findings at the tissue level, we examined the intratumoral distribution of **DCIP‐AcGal** by fluorescence imaging of tumor cryosections. As shown in Figure 6g,h, palbociclib‐treated tumor sections displayed intense **DCIP‐AcGal** fluorescence that was widely distributed throughout the tissue, whereas control tumor sections showed only weak background signals. Overall, these results demonstrated that **DCIP‐AcGal** enabled noninvasive visualization of chemotherapy‐induced tumor senescence in living mice, with high specificity for senescent cells.

### 2.6 Ex vivo imaging of natural brain senescence using DCIP‐AcGal

**FIGURE 7:**
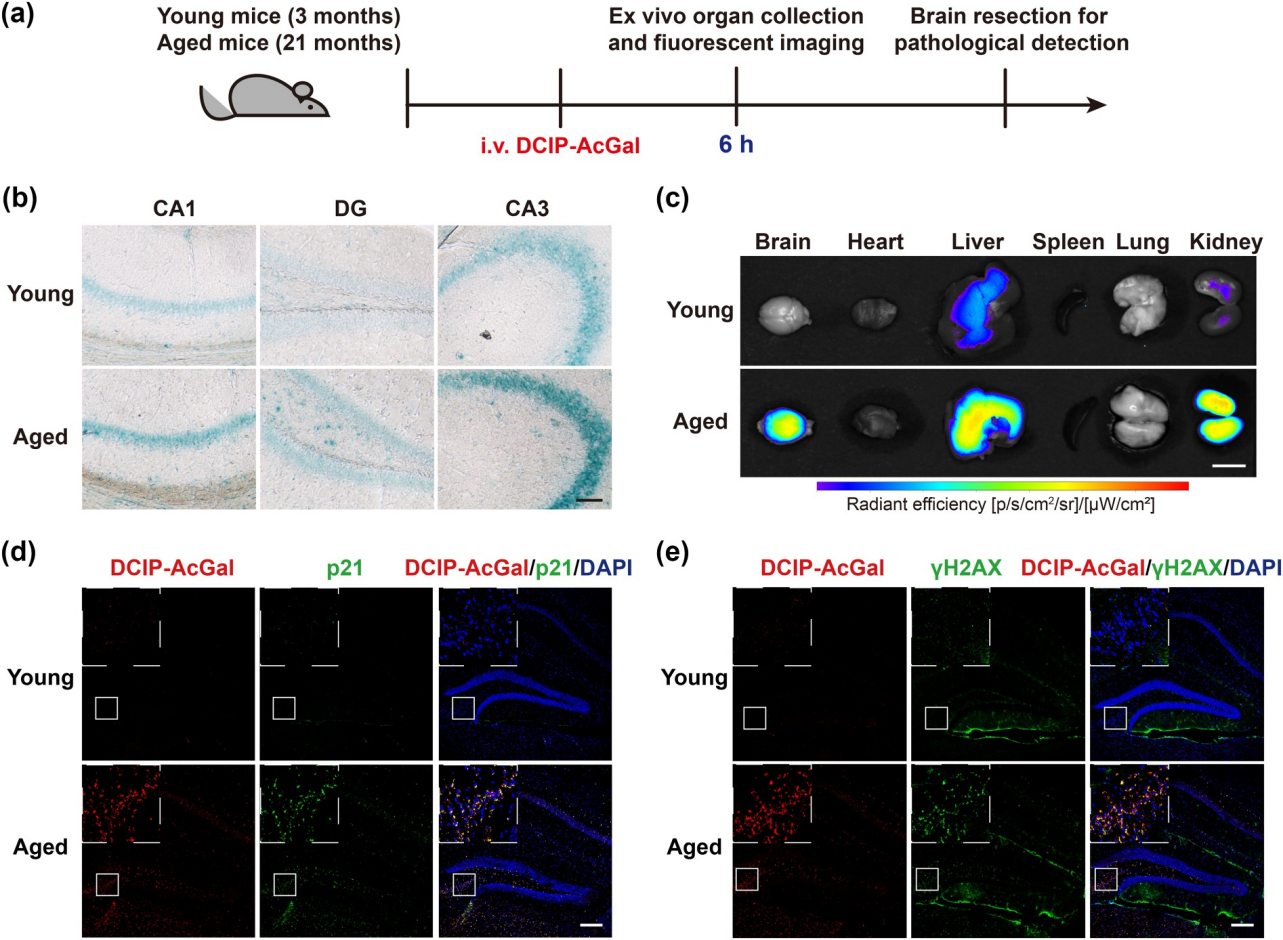
Ex vivo imaging of natural brain senescence using **DCIP‐AcGal**. (a) Schematic illustration of the tumor senescence modeling procedure. (b) X‐gal staining of hippocampal sections for young (3 months) and aged (21 months) mice. Scale bar, 100 μm. (c) Ex vivo fluorescence imaging of major organs including brain, heart, liver, spleen, lung, and kidney, harvested from young and aged mice following intravenous injection of **DCIP‐AcGal** (*λ*_ex_ = 535 nm, *λ*_em_ = 680 nm). Scale bar, 1 cm. (d, e) Immunofluorescence imaging of (d) p21 (green) and (e) γH2AX (green) in the hippocampal cryosections from young and aged mice after intravenous injection of **DCIP‐AcGal** (red), and counterstained with DAPI (blue). *λ*_ex_ = 552 nm, *λ*_em_ = 620–675 nm. Scale bar, 100 μm. DAPI, 4',6‐diamidino‐2‐phenylindole.

Given the critical role of cellular senescence in age‐related neurodegenerative disorders, the ability to non‐invasively assess senescence burden in the brain is of particular importance. The hippocampus, a region essential for learning and memory, is notably susceptible to age‐related senescence accumulation, making it a key target for imaging studies.[^52^, ^53^] Furthermore, the elevated activity of SA‐β‐gal, a hallmark of cellular senescence, has been consistently observed in the aging brain, making it an attractive target for imaging the regional burden and progression of brain senescence.[^54^, ^55^] However, due to the presence of the BBB, few β‐gal‐activatable probes have been able to cross this barrier and enable senescence detection in the brain. To address this gap, we next investigated the applicability of **DCIP‐AcGal** for detecting natural senescence in brain tissue to extend our studies beyond therapy‐induced tumor models. The experimental procedures are illustrated in Figure 7a. To confirm the successful establishment of the natural brain senescence model, we performed X‐gal staining on hippocampal sections in both age groups. As shown in Figure 7b, the hippocampi of aged mouse exhibited deeper blue staining compared to young controls, indicating elevated SA‐β‐gal activity in the aged brain. This observation confirmed the accumulation of senescent cells in the hippocampi during natural aging, validating the model for subsequent imaging studies.

To circumvent the limited penetration depth of NIR fluorescence through the intact skull, we performed ex vivo imaging to evaluate β‐gal activity in the brain. Following intravenous injection of **DCIP‐AcGal**, the brain, heart, liver, spleen, lung, and kidney were harvested from young (3 months) and aged (21 months) mice for ex vivo fluorescence analysis (Figure 7c). Quantification of fluorescence signals revealed that the aged brain exhibited approximately two‐fold higher fluorescence intensity compared to the young brain (Supporting Information S1: Figure S18A). Notably, the liver and kidneys also displayed increased fluorescence in aged mice, likely due to a combination of probe metabolism and the presence of senescent cells in these organs. These results illustrated that **DCIP‐AcGal** enabled sensitive detection of natural brain senescence through specific visualization of SA‐β‐gal activity.

To further characterize the cellular localization of **DCIP‐AcGal** in the aged brain, we performed immunofluorescence staining on hippocampal cryosections from young and aged mice after intravenous injection of **DCIP‐AcGal**. As illustrated in Figure 7d and Supporting Information S1: Figure S18B, the aged group displayed intense **DCIP‐AcGal** fluorescence, whereas the young group showed only minimal signals. Importantly, **DCIP‐AcGal** fluorescence strongly colocalized with two well‐established senescence markers, p21 and γH2AX, as evidenced by the merged images in aged hippocampal neurons (Figure 7d,e). The colocalization of DCIP‐AcGal fluorescence with p21 and γH2AX was particularly noteworthy, as these two markers reflected complementary aspects of the senescent phenotype: p21 upregulation indicated cell‐cycle arrest, while γH2AX marked persistent DNA damage respons. The presence of both markers in cells positive for DCIP‐AcGal provided strong evidence that the probe specifically labeled cells with established senescence features, confirming its reliability as a neuro‐senescence‐associated imaging probe.

To assess the in vivo biosafety of **DCIP‐AcGal**, we performed serum biochemical analysis and hematoxylin and eosin (H&E) staining of major organs following intravenous injection of the probe. As shown in Supporting Information S1: Figure S19A, liver and kidney function markers, including ALT, AST, ALB, ALP, TBIL, GGT, CREA, and BUN, were measured in mice 24 h after intravenous injection of **DCIP‐AcGal** or vehicle control. No statistically significant differences were observed between the two groups for any of the parameters examined, indicating that probe administration did not impair hepatic or renal function. Furthermore, H&E staining of major organs revealed normal histological features in the heart, liver, spleen, lung, and kidney of **DCIP‐AcGal**‐treated mice, with no observable signs of tissue damage, inflammation, or morphological abnormalities compared to the vehicle control group (Supporting Information S1: Figure S19B). These results demonstrated that **DCIP‐AcGal** possessed excellent biocompatibility and biosafety in vivo, with no detectable hepatotoxicity, nephrotoxicity, or organ‐specific toxicity following systemic administration. This favorable safety profile supported the suitability of **DCIP‐AcGal** for in vivo senescence imaging applications.

## 3 CONCLUSIONS

In summary, we have developed a NIR fluorescent probe **DCIP‐AcGal** for the sensitive detection and imaging of senescent cells. The probe features a **DCI**‐derived fluorophore conjugated with an acetylated β‐galactose moiety, enabling specific activation by SA‐β‐gal to restore NIR fluorescence. This design, together with the favorable lipophilicity imparted by the **DCI** core and acetylated modification, facilitates systemic administration and potential BBB permeability. **DCIP‐AcGal** enabled specific imaging of senescent cells, and its activation has been validated by GLB1 knockdown and lysosomal colocalization. Following intravenous injection, the probe successfully visualized therapy‐induced senescence in tumor‐bearing mice and was able to cross the BBB to assess the accumulation of senescent cells in the brains of naturally aging mice. Overall, these findings establish **DCIP‐AcGal** as a powerful tool for investigating senescence biology and monitoring senotherapeutic interventions.^[56]^ Although **DCIP‐AcGal**, operating in the NIR window, has achieved these advances, optical imaging modalities remain limited in terms of tissue penetration depth. For example, in vivo imaging of brain senescence in this study demonstrated that the skull significantly attenuates the fluorescence signal. Given the unique advantages of this lipophilic probe in senescence imaging, a modular click site was introduced to incorporate fluorine, whose isotope fluorine‐18 is a commonly used radiotracer in PET imaging. Future efforts will focus on developing NIR fluorescence‐PET bimodal probes to achieve deeper tissue penetration, thereby enabling non‐invasive in vivo detection of senescence in brain.

## AUTHOR CONTRIBUTIONS

The manuscript was drafted and reviewed through the contributions of all authors. All authors have given approval to the final version of the manuscript.

## CONFLICT OF INTEREST STATEMENT

The authors declare no conflicts of interest.

## ETHICS STATEMENT

All animal experiments were performed according to the guidelines of the Care and Use of Laboratory Animals formulated by the Ministry of Science and Technology of China. All experimental protocols were approved by the Institutional Animal Care and Use Committee of Zhejiang University (ZJU20240651).

## Supporting information

Supporting Information S1

## Data Availability

The data that supports the findings of this study are available in Supporting Information S1 of this article.
