# Supplementary material for: Rational design of a lipophilic β‐galactosidase‐responsive near‐infrared probe for in vivo imaging of cellular senescence
Source: Smart Mol. 2026 Aug 18:e70091. Online ahead of print. doi: 10.1002/smo2.70091 (PMC13487403; doi:10.1002/smo2.70091)
Supplement: Supplementary file 1 [file SMO2-9999-0-s001.docx]

*Supporting Information*

**Rational design of a lipophilic β-galactosidase-responsive near-infrared probe for in vivo imaging of cellular senescence**

*Jiani Huang ^a,b,c,‡^, Feiyi Chu ^e,‡^, Bin Feng ^e^, Lin Chen ^a,b,c^, Peili Cen ^a,b,c^, Jing Wang ^a,b,c^, Ying Qu ^a,b,c^, Lu Hong ^a,b,c^, Fei Wu ^a,b,c^, Huanfeng Tian ^e^, Yanpeng Fang ^e^, Yuanjie Chen ^a,b,c^, Mei Tian ^d,*^, Wenbin Zeng ^e,*^, Hong Zhang ^a,b,c,*^, Yan Zhong ^a,b,c,*^*

a Department of Nuclear Medicine, Zhejiang Cancer Hospital, Hangzhou Institute of Medicine, Chinese Academy of Sciences, Hangzhou, Zhejiang 310022, China
b Institute of Nuclear Medicine and Molecular Imaging of Zhejiang University, Hangzhou, Zhejiang 310009, China
c Key Laboratory of Medical Molecular Imaging of Zhejiang Province, Hangzhou, Zhejiang 310009, China
d Human Phenome Institute, Fudan University, Shanghai, 200040, China
e Xiangya School of Pharmaceutical Sciences, Central South University, Changsha 410013, China

‡ These authors contributed equally to this work.

Correspondence: Yan Zhong, yanzhong@zju.edu.cn; Hong Zhang, hzhang21@zju.edu.cn; Wenbin Zeng, [wbzeng@hotmail.com](mailto:wbzeng@hotmail.com); Mei Tian, tianmei@fudan.edu.cn.

1. **Synthesis of compounds**

Scheme S1. Synthetic route of **DCIP-AcGal**

1.1. Synthesis of **Bz-Gal**

2,4-Dihydroxybenzaldehyde (690 mg, 5 mmol) and K_2_CO_3_ (2.76 g, 6 mmol) were dissolved in acetonitrile (20 mL) and stirred at room temperature for 15 min. Then, 2,3,4,6-tetra-O-acetyl-α-D-galactopyranosyl bromide (2.06 g, 5 mmol) was added to the solution. The mixture was stirred at room temperature for 12 h. The reaction mixture was poured into water and extracted with ethyl acetate three times. The organic layer was dried over anhydrous Na_2_SO_4_ and concentrated under reduced pressure. Purification by column chromatography on silica gel (petroleum ether/ethyl acetate, 1:2, v/v) afforded **Bz-Gal** (1.4 g, 60%) as a white solid.

1.2. Synthesis of **DCI**

To a solution of isophorone (5.52 g, 40 mmol) in ethanol (50 mL) were added malononitrile (5.28 g, 80 mmol) and piperidine (0.5 mL) dropwise. The mixture was refluxed at 80℃ under nitrogen for overnight. After the reaction was cooled to room temperature, the mixture was concentrated under reduced pressure. Purification by column chromatography on silica gel (petroleum ether/dichloromethane, 10:1, v/v) afforded **DCI** (2.98 g, 40%) as a white crystal.

1.3. Synthesis of **DCIO-AcGal**

To a solution of **DCI** (558 mg, 3 mmol) and **Bz-Gal** (936 mg, 2 mmol) in ethanol (20 mL), piperidine (2 drops) was added. The mixture was refluxed at 90℃ under nitrogen for 4 h. After cooling to room temperature, the reaction mixture was poured into water and extracted with dichloromethane three times. The organic layer was dried over anhydrous Na_2_SO_4_ and concentrated under reduced pressure. Purification by column chromatography on silica gel (dichloromethane/methanol, 100:1, v/v) afforded **DCIO-AcGal** (573 mg, 45%) as an orange solid.^1^H NMR (500 MHz, CDCl_3_) δ 7.59 (s, 1H), 7.45 (d, J = 9.0 Hz, 1H), 7.34 (d, J = 16.2 Hz, 1H), 7.02 (d, J = 16.2 Hz, 1H), 6.78 (s, 1H), 6.59 (d, J = 7.3 Hz, 2H), 5.46 (dd, J = 10.7, 7.1 Hz, 2H), 5.15 (dd, J = 15.3, 5.4 Hz, 2H), 4.26 (dd, J = 13.9, 9.3 Hz, 1H), 4.14 (t, J = 5.3 Hz, 2H), 2.57 (s, 2H), 2.48 (s, 2H), 2.16 (s, 3H), 2.10 (s, 3H), 2.07 (s, 3H), 2.03 (s, 3H), 1.07 (s, 6H).^13^C NMR (126 MHz, CDCl_3_) δ 170.74, 170.34, 170.28, 169.78, 169.73, 158.69, 156.43, 155.39, 131.99, 129.06, 128.26, 122.67, 118.69, 113.75, 113.05, 109.18, 104.78, 98.89, 71.08, 70.80, 68.72, 66.93, 61.34, 60.60, 43.05, 39.10, 31.99, 27.97, 20.73, 20.66, 20.55, 20.52.

1.4. Synthesis of **DCIA-AcGal**

To a solution of **DCIO-AcGal** (606 mg, 1 mmol) in acetonitrile (20 mL) were added propargyl bromide (353.7 mg, 3 mmol) and K₂CO₃ (414 mg, 3 mmol). The mixture was refluxed at 70°C under nitrogen for 4 h. After cooling to room temperature, the reaction mixture was poured into water and extracted with dichloromethane three times. The organic layer was dried over anhydrous Na₂SO₄ and concentrated under reduced pressure. Purification by column chromatography on silica gel (dichloromethane/methanol, 20:1, v/v) afforded **DCIA-AcGal** (417 mg, 50% ) as an orange solid. ^1^H NMR (400 MHz, CDCl_3_) δ 7.51 (d, J = 8.7 Hz, 1H), 7.33 (d, J = 16.2 Hz, 1H), 6.99 – 6.92 (d, 1H), 6.83 – 6.79 (s, 1H), 6.74 (d, J = 2.1 Hz, 1H), 6.68 (dd, J = 8.6, 2.0 Hz, 1H), 5.53 – 5.48 (m, 1H), 5.47 (s, 1H), 5.17 – 5.12 (m, 1H), 5.11 (d, J = 7.7 Hz, 1H), 4.78 (d, J = 2.2 Hz, 2H), 4.25 (dd, J = 11.3, 6.8 Hz, 1H), 4.18 (dd, J = 11.3, 6.2 Hz, 1H), 4.10 (t, J = 6.6 Hz, 1H), 2.61 (t, J = 2.1 Hz, 1H), 2.59 (s, 2H), 2.48 (s, 2H), 2.19 (s, 3H), 2.08 (s, 3H), 2.07 (s, 3H), 2.02 (s, 3H), 1.08 (s, 6H). ^13^C NMR (126 MHz, CDCl_3_) δ 170.28, 170.14, 170.00, 169.41, 169.36, 158.91, 156.70, 154.82, 131.05, 128.60, 128.42, 123.02, 120.53, 113.69, 112.95, 109.49, 102.50, 99.07, 77.88, 77.72, 76.63, 71.20, 70.67, 68.46, 66.88, 61.41, 56.36, 42.97, 39.02, 31.99, 26.85, 20.72, 20.68, 20.60, 20.54.

1.5. Synthesis of **DCIP-AcGal**

**F-Az** was prepared according to the previous method^[1]^. To a solution of 1-fluoro-2-iodoethane (348 mg, 2 mmol) in DMF (4 mL) was added sodium azide (390 mg, 6 mmol). The reaction mixture was stirred at room temperature in the dark overnight. After completion as monitored by GC-MS, stirring was stopped and the mixture was allowed to stand for precipitation. The supernatant was filtered to afford a DMF solution of the crude product, which was used directly in the next step.

To a solution of **DCIA-AcGal** (35 mg, 0.052 mmol) and **F-Az** (4.6 mg, 0.052 mmol) in DMF (5 mL) was added dropwise a freshly prepared mixture of copper(II) sulfate (1 mg) and sodium ascorbate (2 mg) in deionized water (1 mL). The resulting mixture was stirred at 50°C for 30 min. After completion, as monitored by TLC, the reaction mixture was poured into water and extracted with ethyl acetate three times. The organic layer was dried over anhydrous Na_2_SO_4_ and concentrated under reduced pressure. Purification by column chromatography on silica gel (petroleum ether/ethyl acetate, 3:1, v/v) afforded compound **DCIP-AcGal** (341 mg, 50% ) as an orange solid.^1^H NMR (500 MHz, DMSO-*d_6_*) δ 8.33 (s, 1H), 7.78 (d, *J* = 8.8 Hz, 1H), 7.37 – 7.28 (m, 2H), 6.92 (d, *J* = 1.9 Hz, 1H), 6.76 (s, 1H), 6.69 (dd, *J* = 8.7, 1.9 Hz, 1H), 5.63 (d, *J* = 7.3 Hz, 1H), 5.38 (d, *J* = 2.7 Hz, 1H), 5.30 (t, *J* = 4.4 Hz, 2H), 5.28 – 5.24 (m, 2H), 4.89 (t, *J* = 4.6 Hz, 1H), 4.80 – 4.77 (m, 2H), 4.74 – 4.72 (m, 1H), 4.50 (t, *J* = 6.3 Hz, 1H), 4.12 (t, *J* = 5.9 Hz, 2H), 2.59 (s, 2H), 2.46 (s, 2H), 2.16 (s, 3H), 2.07 (s, 3H), 2.00 (s, 3H), 1.97 (s, 3H), 1.00 (s, 6H). ^13^C NMR (126 MHz, DMSO-*d_6_*) δ 170.72, 170.45, 170.36, 170.04, 169.73, 159.15, 157.99, 156.74, 143.10, 131.95, 129.64, 129.02, 125.42, 122.58, 120.08, 114.46, 113.62, 109.29, 102.71, 97.53, 83.04, 81.71, 76.05, 71.03, 70.65, 68.64, 67.74, 62.72, 61.96, 42.79, 38.51, 32.11, 27.84, 20.98, 20.93, 20.87, 20.82. HRMS: theoretical for C_38_H_42_N_5_O_11_F [M+H]^+^, 764.2943; found, 764.2947.

**2. Materials and methods**

2.1. Materials and apparatus

All chemicals were of analytical reagent grade and applied without further refinement. All solutions and buffers were prepared using deionized water that has been passed through a water ultra-purification system. A Bruker AV-500 spectrometer (Rheinstetten, Germany) was used to obtain the ^1^H NMR spectra and ^13^C NMR spectra. High-resolution mass spectra (HRMS) were measured on a Thermo Scientific instrument. High-performance liquid chromatography (HPLC) analysis was performed on an Agilent 1100 system with a C8 column (American). A UV-2450 UV-Vis spectrophotometer (Shimadzu, Japan) was used to measure the UV-Vis spectra. A Hitachi F-2700 fluorescence spectrophotometer (Hitachi, Japan) was employed to record the fluorescence spectra with slits of 10 nm excitation and 10 nm emission. All these processes were implemented at 25°C using standard quartz cuvettes with a 10-mm light path.

2.2. General procedure for β-galactosidase (β-gal) detection

Unless otherwise noted, the fluorescence of **DCIP-AcGal** (10 μM) reacting with β-gal was determined in DMSO/PBS (3:7, v/v, pH 7.4). The stock solution (10 mM) of **DCIP-AcGal** was prepared by dissolving it in DMSO. In test tubes, 989 μL of DMSO/PBS (299 μL + 690 μL) and 1 μL of the stock solution of **DCIP-AcGal** were mixed, followed by adding β-gal solution (10 μL, 500 U/mL in PBS). After incubation at 37°C for 30 min, the portion of the reaction solution was transferred to a quartz cell to measure the UV-Vis absorption spectra and fluorescence emission spectra (λ_ex_ = 540 nm). At the same time, a control solution without β-gal was prepared and measured under the same conditions for comparison.

2.3. Limit of detection

The detection limit was calculated based on the fluorescence titration. In the absence of β-gal, the fluorescence emission spectra of **DCIP-Gal** were measured six times, and the standard deviation of blank measurement was achieved. To gain the slope, the fluorescence intensity at F_657_ was plotted against the concentration of β-gal.

The detection limit was calculated with the following equation:

Detection limit = 3σ/k

wherein σ is the standard deviation of blank measurement, and k is the slope between the F_611_ versus β-gal concentration.

2.4. Methods of Theoretical Calculations

Calculations of optimal geometries and electronic structures of **DCIP-AcGal** were carried out by density functional theory (DFT) at the B3LYP/6-31+G(D) level^[2]^. The starting structures with different conformational isomers were considered to ensure that the optimized structure corresponds to a global minimum. All these calculations were carried out with Gaussian 16 software. The obtained data were analyzed by GaussView 5.0 software.

2.5. Molecular Dynamics simulations

Molecular Dynamics (MD) simulations were performed using GROMACS 2023.1 with the CHARMM36 all-atom force field to investigate membrane interactions of **DCIP-AcGal** and **DCIP-Gal** ^[3]^. The bilayer membrane model (422 POPC molecules) was constructed via CHARMM-GUI. The system was solvated in TIP3P water and neutralized with 0.15 M NaCl to mimic physiological salinity. Energy minimization employed steepest descent followed by conjugate gradient algorithms. Subsequent equilibration was conducted under NVT and NPT ensembles. Temperature coupling used the V-rescale algorithm $\tau=0.1ps$. Pressure coupling employed the Berendsen barostat during pre-equilibration and Parrinello-Rahman barostat (compressibility 4.5×10^-5^ bar^-1^) during production. Production runs utilized the leap-frog integrator (2-fs timestep). Long-range electrostatics were treated with PME, and non-bonded interactions had a 1.2-nm cutoff. All bonds were constrained via LINCS. Trajectory analysis employed GROMACS utilities, MDAnalysis, and MDTraj, with visualization conducted in VMD and PyMOL.

2.6. Predictions of blood-brain barrier (BBB) penetration

Three established computational platforms (ADMETlab3.0, DrugFlow, and DeepPK) provide the predicted probability that a compound belongs to the BBB-permeable class^[4-6]^. These values range from 0 to 1, with higher values indicating a greater likelihood of BBB penetration. In addition to this probability output, DeepPK also reports a second BBB-related endpoint, logPS, which represents the logarithm of the permeability-surface area product and reflects the extent of compound transport across the BBB in vivo. For this endpoint, a higher value, meaning a less negative logPS value, indicates greater BBB permeability. Because these outputs differed in scale and interpretation, the effect of acetylation was evaluated according to the nature of each endpoint. For the probability-based outputs, the change was expressed as the ratio of the post-acetylation value to the pre-acetylation value. For the logPS output, the shift on the logarithmic scale was converted into the corresponding change on the linear permeability scale, so that all readouts could be presented within a common fold-change framework while retaining the meaning of the original model output.

2.7. Selectivity and competitive experiments

Stock solutions (10 μM) of testing species including ions (Na_2_CO_3_, FeSO_4_, FeCl_3_, KH_2_PO_4_, MgCl_2_, CaCl_2_), amino acids (Cys, Hcy, GSH), enzymes (ALP, GGT, CE, NTR, APN, Try, Chymotrypsin), and bioactive substances (H_2_O_2_, HClO, BSA, Heparin, glucose, ribose) and dissolved in PBS were prepared, and then further diluted with a DMSO/PBS (3/7, v/v, 10 μM, pH = 7.4) mixed solution to prepare a final concentration of 100 μM and kept at 4°C in a refrigerator. The fluorescence intensity ratio was calculated after 22 min at 37℃, upon addition of β-gal at excitation of 657 nm.

2.8. Cell culture

Human breast cancer (MDA-MB-231), human colon cancer (HCT116), mouse hippocampal neuronal (HT22), and human embryonic lung fibroblast (IMR-90) cell lines were purchased from Zhejiang Meisen Cell Technology Co., Ltd. MDA-MB-231, HT22, and IMR-90 cells were cultured in high-glucose DMEM supplemented with 10% fetal bovine serum (FBS) and 1% penicillin-streptomycin. HCT116 cells were cultured in RPMI-1640 medium supplemented with 10% FBS and 1% penicillin-streptomycin. All cells were maintained at 37°C in a humidified incubator with 5% CO_2_.

2.9. Induction and validation of cellular senescence

To induce senescence, MDA-MB-231 and HCT116 cells were treated with doxorubicin (1 mg/mL) for 2 h, followed by culture in fresh complete medium for 3 days. HT22 cells were treated with bleomycin (10 μM) for 24 h. IMR-90 cells at population doubling level 50 (PDL50) were used as replicative senescent cells, with PDL5 cells serving as controls.

2.10. Cell viability

Normal or senescent cells were seeded in 96-well plates at 4×10^4^ cells per well and incubated overnight. Afterwards, cells were treated with various concentrations of **DCIP-AcGal** (0, 2.5, 5, 10, 25, and 50 μM) for 24 h. Cell viability was assessed using the CCK-8 assay according to the manufacturer’s protocol.

2.11. Western Blotting

Proteins were extracted using RIPA lysis buffer (Beyotime) containing 1% protease and phosphatase inhibitor cocktail (Beyotime). Protein concentrations were determined by BCA assay (ThermoFisher). Equal amounts of protein were separated by 4-20% SDS-PAGE electrophoresis and transferred onto PVDF membranes. Membranes were blocked with 5% BSA for 1 h at room temperature, and incubated overnight at 4°C with primary antibodies including β-actin (1:1000, CST) and GLB1/β-galactosidase (1:10000, Abcam). Subsequently, membranes were incubated with appropriate horseradish peroxidase (HRP)-conjugated secondary antibodies (1:10000, CST) for 1 h at room temperature. Protein bands were detected using ECL reagents and imaged with a gel imaging system (Shenhua, China). The gray value of protein bands was analyzed with Image J software, with β-actin as the control.

2.12.Senescence-associated β-galactosidase (SA-β-gal) staining

SA-β-gal staining was performed using a commercial kit (Beyotime). Cells or tissue sections were fixed and incubated overnight at 37°C (without CO_2_) using staining solution, and then observed using brightfield microscopy (Zeiss, Germany). Blue stained cells were identified as senescent.

2.13. Cell fluorescence imaging

Cells were seeded on glass coverslips in 24-well plates at 2×10^5^ cells per well. After treatment with 10 μM **DCIP-AcGal** for 24 h, nuclei were stained with Hoechst 33342 for 10 min. Images were captured using a confocal microscope (Leica, Germany).

2.14. Flow cytometry

Cells were seeded in 6-well plates at 8×10^5^ cells per well and treated with 10 μM **DCIP-AcGal** for 24 h. Cells were then collected and analyzed by flow cytometry (Beckman, America). Data were processed using FlowJo software.

2.15. GLB1 knockdown

siRNA targeting GLB1 (5’-CUACACAAAUCAGCGAUUU-3’) and scrambled control siRNA (5’-CCUACGCCACCAAUUUCGU-3’) were synthesized by Shanghai GenePharma Co., Ltd. Transfection was performed using siRNA-mate plus transfection reagent (GenePharma, China) according to the manufacturer’s instructions^[7]^.

2.16. RNA extraction and reverse transcription quantitative polymerase chain reaction (RT-qPCR)

Total RNA was extracted using TRIzol (Invitrogen, America). cDNA was synthesized using Evo M-MLV RT Premix (Accurate Biotech, China). RT-qPCR was performed using SYBR Green Premix Pro Taq HS qPCR Kit (Accurate Biotech, China) on a Bio-Rad CFX-96 system. Cycling conditions were: 95°C for 30 s, followed by 40 cycles of 95°C for 5 s and 60°C for 30 s. Relative gene expression was normalized to β-actin. Primer sequences were as follows: GLB1 forward, 5’-TATACTGGCTGGCTAGATCACTG-3’, reverse, 5’-GGCAAAATTGGTCCCACCTATAA-3’; β-actin forward, 5’-CATGTACGTTGCTATCCAGGC-3’, reverse, 5’-CATGTACGTTGCTATCCAGGC-3’.

2.17. Colocalization analysis

Senescent cells treated with 10 μM **DCIP-AcGal** for 24 h were stained with Lyso-Tracker Green (Beyotime) for 30 min and Hoechst 33342 for 10 min. Fluorescent images were obtained by confocal microscopy and analyzed using Image J software.

2.18. Cell immunofluorescence staining

Cells treated with 10 μM **DCIP-AcGal** for 24 h were fixed with 4% paraformaldehyde (PFA), blocked with 5% BSA, and incubated with anti-p21 antibody (1:200, Abcam) overnight at 4°C. Then, cells were incubated with fluorescent secondary antibodies (1:1000, Abcam) for 1 h and DAPI for 5 min at room temperature. Images were acquired using a confocal microscope.

2.19. Tumor senescence model construction and validation

All animal experiments were approved by the Institutional Animal Care and Use Committee of Zhejiang University (ZJU20240651). Female BALB/C nude mice (6-8 weeks old) were purchased from Shanghai SLAC Laboratory Animal Co., Ltd. A subcutaneous xenograft tumor model was established by inoculating MDA-MB-231 cells (1×10^7^) suspended in matrigel into the second mammary fat pad on the right side of nude mice. When tumor volume reached approximately 100 mm^3^, the body weights of mice and tumor volumes were measured every other day. Tumor volume was calculated using the formula: Volume = length (mm) × width (mm) × width (mm) × 0.5. To induce senescence, mice were administered palbociclib (100 mg/kg in 50 mM sodium lactate, pH 4.0) daily by gavage for 14 days, while the control group was treated with solvents. Tumors collected at the treatment endpoint were sectioned, and subjected to SA-β-gal staining or Ki67 immunohistochemical staining to verify the tumor cellular senescence.

2.20. Natural brain senescence model construction and validation

Male C57BL/6J mice aged 3 and 21 months were obtained from Shanghai SLAC Laboratory Animal Co., Ltd. Brain tissues were collected and processed for SA-β-gal staining to assess cellular senescence.

2.21. Ki67 immunohistochemical staining

The immunohistochemical staining for Ki67 was performed on paraffin-embedded tissue sections. Briefly, sections were dewaxed in xylene and rehydrated through a graded ethanol series. Antigen retrieval was carried out in EDTA buffer (pH 9.0) using a microwave oven. Endogenous peroxidase activity was blocked with 3% H₂O₂. After blocking with 3% BSA, sections were incubated overnight at 4°C with a primary antibody against Ki67 (HUABIO, 1:1000 dilution). Subsequently, sections were incubated with an HRP-conjugated secondary antibody, and the signal was visualized using a DAB substrate. Nuclei were counterstained with hematoxylin. Finally, sections were dehydrated, cleared, and examined by a digital pathology scanner (KFBIO, China).

2.22. In vivo and ex vivo fluorescence imaging

Mice were injected with **DCIP-AcGal** (4 mM, 100 μL in DMSO:Cremophor EL:saline = 1:1:18) via the tail vein. For in vivo fluorescence imaging, mice were anesthetized with isoflurane, and images were acquired at different time points using an In Vivo Imaging System (Biolight Biotech, China) with excitation at 530 nm and emission at 690 nm. Mice were sacrificed, and the hearts, livers, spleens, lungs, kidneys, and tumors/brains were collected for ex vivo fluorescence imaging.

2.23. Tissue immunofluorescence imaging

Tumor and brain tissues were fixed in 4% PFA and dehydrated in 30% sucrose. Subsequently, frozen sections (thickness, 30 μm) were prepared, then blocked and permeabilized in 5% BSA (in PBS containing 0.3% Triton X-100) for 1 h. Sections were incubated with primary antibodies overnight at 4°C: p21 antibody (abcam, 1:200 dilution), γH2AX antibody (CST, 1:200 dilution). After incubation with secondary fluorescent antibodies for 1 h and DAPI for 5 min at room temperature, images were obtained by confocal microscopy and analyzed using Image J software.

2.24. Safety evaluation

Mice were injected with **DCIP-AcGal** or vehicle. After 1 day, blood samples were collected, and serum was isolated for biochemical analysis of ALT, AST, ALB, ALP, TBIL, GGT, CREA, and BUN. Major organs (hearts, livers, spleens, lungs, kidneys) were fixed, sectioned, and stained with hematoxylin and eosin (H&E) for histological evaluation. Sections were scanned using a digital pathology scanner.

2.25. Statistical analysis

Data were expressed as mean ± standard error of mean (SEM). Differences between two groups were evaluated by a two-tailed unpaired Student’s t-test, with *P* < 0.05 indicating statistical significance. Statistical significance is indicated in the figures by ns (not significant), **P* < 0.05, ***P* < 0.01, ****P* < 0.001, and *****P* < 0.0001.

**Figures**

Scheme S2. Direct activation pathway and esterase-mediated cascade activation pathway of **DCIP-AcGal** by β-gal.

Figure S1. ^1^H-NMR spectrum of **DCIO-AcGal**.

Figure S2. ^13^C-NMR spectrum of **DCIO-AcGal**.

Figure S3. ^1^H-NMR spectrum of **DCIA-AcGal**.

Figure S4. ^13^C-NMR spectrum of **DCIA-AcGal**.

Figure S5. ^1^H-NMR spectrum of **DCIP-AcGal**.

Figure S6. ^13^C-NMR spectrum of **DCIP-AcGal**.

C_38_H_42_N_5_O_11_F [M+H]^+^ calcd: 764.2943; found, 764.2947.

Figure S7. HR-MS spectrum of **DCIP-AcGal**.

Figure S8. Photostability of **DCIP-AcGal** (10 μM) before and after incubated with β-gal (2 U/mL) for 45 min.

Figure S9. Fluorescence emission spectra of **DCIP-AcGal** (10 μM) in the absence or presence of D-Gal (0.1 mM or 1 mM) before and after incubation with β-gal (2 U/mL).

Figure S10. HRMS of **DCIP-AcGal** (10 μM) incubated with β-gal (2U/mL) for 45 min.

Figure S11. Distance of **DCIP-AcGal** to the bilayer center during molecular dynamics simulations.

Figure S12. Distance of **DCIP-Gal** to the bilayer center during molecular dynamics simulations.

Figure S13. RMSD of **DCIP-AcGal** during molecular dynamics simulations.

Figure S14. RMSD of **DCIP-Gal** during molecular dynamics simulations.

Figure S15. Cell viabilities of control or senescent (A) MDA-MB-231, (B) HCT116, (C) HT22 and (D) IMR-90 cells after incubation with **DCIP-AcGal** for 24 h (n = 4).

Figure S16. (A) Flow cytometric analysis of **DCIP-AcGal** uptake in control and senescent MDA-MB-231 and HCT116 cells. (B) Semiquantitative analysis of mean fluorescence intensity (MFI) in (A) (n = 3). (Statistical significance was determined using a two-tailed unpaired Student’s t-test. *****P* < 0.0001)

Figure S17. (A) Ex vivo fluorescence imaging of major organs (heart, liver, spleen, lung, kidney) and tumors harvested from control and palbociclib-treated mice at 30 min post-injection of **DCIP-AcGal**. (B) Quantification of tumor fluorescence intensities in (A) (n = 3). (Statistical significance was determined using a two-tailed unpaired Student’s t-test. **P* < 0.05)

Figure S18. (A) Quantification of brain fluorescence intensity from ex vivo imaging (n = 3). (B) Semiquantitative analysis of **DCIP-AcGal** fluorescence intensity in hippocampus cryosections from young and aged mice after intravenous injection with **DCIP-AcGal**. (Statistical significance was determined using a two-tailed unpaired Student’s t-test. ****P* < 0.001)

Figure S19. In vivo biosafety evaluation of **DCIP-AcGal**. (A) Serum biochemical analysis of liver and kidney function in mice 24 h after intravenous injection of **DCIP-AcGal** or vehicle control, including ALT, AST, ALB, ALP, TBIL, GGT, CREA, and BUN (n = 5). (B) Representative H&E staining of major organs (heart, liver, spleen, lung, and kidney) harvested from mice 24 h after intravenous injection of **DCIP-AcGal** or vehicle control. Scale bar, 200 μm.

**References**

1. Depke, D. A.; Konken, C. P.; Rösner, L.; Hermann, S.; Schäfers, M.; Rentmeister, A., *Chem. Commun.* **2021,** *57*, 9850.

2. Zhou, J.; Feng, B.; Li, C.; Chu, F.; Mo, Y.; Lin, M.; Zhao, Y.; Zhou, J.; Deng, X.; Meng, Q.; Huang, J.; Zhang, H.; Feng, X.; Zeng, W., *Biosens. Bioelectron.* **2025,** *289*, 117862.

3. Fang, Y.; Fan, D.; Feng, B.; Zhu, Y.; Xie, R.; Tan, X.; Liu, Q.; Dong, J.; Zeng, W., *Bioact. Mater.* **2025,** *50*, 510.

4. Fu, L.; Shi, S.; Yi, J.; Wang, N.; He, Y.; Wu, Z.; Peng, J.; Deng, Y.; Wang, W.; Wu, C.; Lyu, A.; Zeng, X.; Zhao, W.; Hou, T.; Cao, D., *Nucleic Acids Res.* **2024,** *52*, W422.

5. Myung, Y.; de Sá, A. G. C.; Ascher, D. B., *Nucleic Acids Res.* **2024,** *52*, W469.

6. Shen, C.; Song, J.; Hsieh, C.-Y.; Cao, D.; Kang, Y.; Ye, W.; Wu, Z.; Wang, J.; Zhang, O.; Zhang, X.; Zeng, H.; Cai, H.; Chen, Y.; Chen, L.; Luo, H.; Zhao, X.; Jian, T.; Chen, T.; Jiang, D.; Wang, M.; Ye, Q.; Wu, J.; Du, H.; Shi, H.; Deng, Y.; Hou, T., *J. Chem. Inf. Model.* **2024,** *64*, 5381.

7. Koo, S.; Won, M.; Li, H.; Kim, W. Y.; Li, M.; Yan, C.; Sharma, A.; Guo, Z.; Zhu, W. H.; Sessler, J. L.; Lee, J. Y.; Kim, J. S., *Chem Sci* **2021,** *12*, 10054.
